# Supplementary figures and images for: ONCOBREAST-TEST Is a Quick Diagnostic, Prognostic and Predictive Method of Response to Systemic Treatment
Source: Cancers (Basel). 2023 Dec 26;16(1):120. doi: 10.3390/cancers16010120 (PMC10778340; doi:10.3390/cancers16010120)

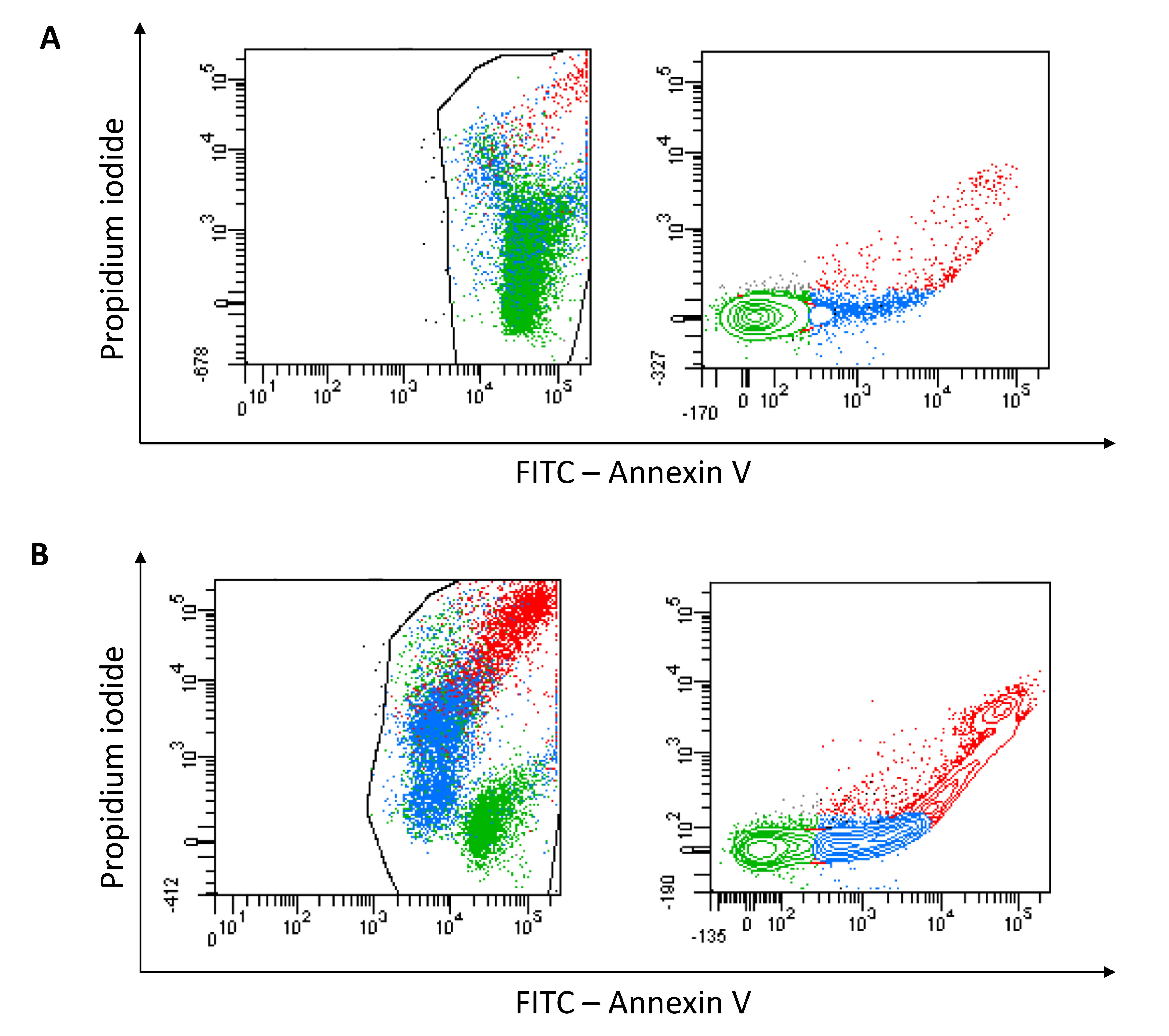

Supplement: Supplementary file 1 [file cancers-16-00120-s001.zip › cancers-2733075-supplementary/cancers-2733075-supplementary/Supl/Figure S1.tiff]
